# Supplementary material for: Survival of glioblastoma treated with a moderately escalated radiation dose—Results of a retrospective analysis
Source: PLoS One. 2020 May 15;15(5):e0233188. doi: 10.1371/journal.pone.0233188 (PMC7228055; doi:10.1371/journal.pone.0233188)
Supplement: S1 Table — (DOCX) [file pone.0233188.s001.docx]

S1 Table Final models using the least absolute shrinkage and selection operator method

| Progression-free survival model | | | Overall survival model | | |
| --- | --- | --- | --- | --- | --- |
| Variable entry | Parameter estimate | AICC^a^ | Variable entry | Parameter estimate | AICC |
| Age group | 0.16401 | 519.688 | Year of diagnosis | 0.39966 | 514.188 |
| Year of diagnosis | 0.11885 | 519.688 | Radiation dose | -0.0975 | 517.639 |
| Radiation volume | -0.0344 | 520.964 | Radiation volume | -0.0087 | 517.639 |

^a^Specifies a small-sample bias-corrected version of Akaike’s information criterion with a correction (AICC) for small sample size.
