# Supplementary material for: Survival of glioblastoma treated with a moderately escalated radiation dose—Results of a retrospective analysis
Source: PLoS One. 2020 May 15;15(5):e0233188. doi: 10.1371/journal.pone.0233188 (PMC7228055; doi:10.1371/journal.pone.0233188)
Supplement: Attachment — Submitted filename: PONE-D-20-02836 review markup.pdf [file pone.0233188.s003.pdf]

Manuscript

Click here to download Manuscript
GBM_PONE_20200131.docx

Survival of glioblastoma treated with a moderately escalated radiation dose – Results of a retrospective analysis

Short title: Glioblastoma treated with a moderately escalated radiation dose

Li-Tsun Shieh[1], How-Ran Guo[2,3], Li-Ching Lin[4], Chin-Hong Chang[5], Sheng-Yow Ho[4,6*]

[1]Department of Radiation Oncology, Chi Mei Medical Center, Liouying, Tainan, Taiwan

[2]Department of Environmental and Occupational Health, College of Medicine, National Cheng Kung University, Tainan, Taiwan

[3]Department of Occupational and Environmental Medicine, National Cheng Kung University Hospital, Tainan, Taiwan

[4]Department of Radiation Oncology, Chi Mei Medical Center, Tainan, Taiwan

[5]Department of Neurosurgery, Chi Mei Medical Center, Tainan, Taiwan

[6]Graduate Institute of Medical Science, Chang Jung Christian University, Tainan, Taiwan

*Corresponding author: Sheng-Yow Ho

e-mail: shengho@seed.net.tw (SYH)

Abstract

Glioblastoma (GBM) has the highest fatality rate among primary malignant brain tumor and typically tends to recur locally just adjacent to the original tumor site following surgical resection and adjuvant radiotherapy. We conducted a study to evaluate the survival outcomes between a standard dose (≤ 60-Gy) and moderate radiation dose escalation (>60-Gy), and to identify prognostic factors for GBMs patients. We retrospectively reviewed the medical records of primary GBM patients who were diagnosed between 2005 and 2016 in two referral hospitals in Taiwan. They were identified from the cancer registry database and followed up from the date of diagnosis to October 2018. The progression-free survival (PFS) and overall survival (OS) were compared between the two dose groups, and independent factors for survival were also analyzed through Cox regression. 142 GBM patients were identified from our cancer registry database, and 84 of them fit the inclusion criteria. Of 84 patients, 52 (62%) were ~~man~~. The radiation dose ranged from 50.0 Gy to 66.6 Gy. Fifteen (18%) patients received an escalated dose boost >60.0 Gy. The escalated group had a longer median PFS (15.4 *vs*. 7.9 months, p =0.01 for log-rank test) than the reference group (with dosage ≤ 60-Gy), and the median OS was also longer in the escalation group (33.8 *vs*. 12.5 months, p <0.01 for log-rank test), respectively. After a multivariate analysis affecting PFS and OS, only the factor of escalated dose was identified as a significant predictor for good prognosis (PFS: hazard ratio [HR] =0.43, p =0.03; OS: HR =0.40, p < 0.01). A moderate radiation dose escalation (> 60 Gy) was found to be associated with an improved PFS and OS in GBM patients. However, some bias cannot be excluded in the study without molecular data. Prospective studies including molecular information are warranted.

Introduction

Glioblastoma (GBM) is the most common central nervous system tumor in adults and the deadliest primary malignant brain tumor. Patients succumb to death shortly after diagnosis, despite multi-modal therapy. Surgical resection of the tumor followed by concurrent chemo-radiotherapy is the standard treatment nowadays. Management of GBM has advanced little in the last two decades. The overall 2-year survival of GBM following optimal treatment is only 27% [1, 2] GBM typically occurs in a single brain lesion and tends to recur locally just adjacent to the original tumor site following resection and adjuvant radiotherapy. According to previous studies on the pattern of failure for GBM patients, more than 80% of tumor relapse occurred within the initial tumor margin [3-5]. Given the importance of local control, it is important to achieve gross tumor resection (GTR) and an escalated radiation dose to avoid local tumor recurrence. Previous analysis from a National Cancer Database revealed those that received GTR do have an increased survival rate over patients that underwent subtotal resection or biopsy only [6]. However, completion of GTR would depend on the risk of neurologic deficits.

In the era of conformal and precise radiation, several studies investigated an escalated radiation dose to the GBM tumor surgical bed or residuum to improve local control [5, 7-12]. Milano et al. investigated the pattern and timing of GBMs treated by biopsy or resection following standard 60-Gy radiation and temozolomide (TMZ) therapy. It was found 80% of recurrence still occurred in the primary tumor site [7]. Several previous studies examined an escalated radiation dose (> 60 Gy), or even higher radiation dose up to 80-90 Gy with the aim to achieve better local control [5, 8-10]. Most of them failed to prolong survival through an escalated dose. However, two recent reports showed a positive prognostic effect on a moderately integrated radiation

boost dose (> 60 Gy) to have a superior local control, and this also translated to a better overall survival (OS) [11, 12].

Given the controversial clinical results, this study aimed to compare survival outcome between the escalated radiation dose (> 60 Gy) and standard radiation dose (≤ 60 Gy) in GBM patients undergoing post-operative adjuvant therapy. Prognostic factors associated with the survival of GBMs were also evaluated.

Materials and Methods

Study population

We retrospectively analyzed primary brain GBM patients in a medical center and its affiliated Liouying branch, both in Tainan, Taiwan. Patients with histology codes ICD-O-3 9440/3 from the cancer registry database during 2005–2016 were identified. GBM variants of gliosarcoma, giant-cell, or epithelioid histologic differentiation were excluded. To be eligible, GBM patients were enrolled with the criteria of older than 20 years old, good performance status (ECOG less than 2), and undergoing curative brain irradiation. We excluded patients who had no pathologic confirmation or had received brain irradiation before the diagnosis GBM. We extracted following information from the database: age, sex, histology, extents of surgery, radiation dose, prescription of chemotherapy, and the date of last follow-up. The follow-up time was from the date of GBM diagnosis to October 2018.

The extent of surgery was assessed by both post-operative image and operation records. All of the brain images were reviewed by an oncologist and a radiologist to confirm the size and location of the brain lesion. All patients received radiation using the technique of intensity-modulated radiation therapy (IMRT). The radiation dose 45–46 Gy was prescribed in 1.8–2 Gy daily fractions to the first planning target volume (PTV), which was defined as the gross tumor volume (GTV) and perifocal edema for

possible microscopic extension. The clinical target volume (CTV) was defined as GTV plus a 1.5–2 cm margin for microscopic extension, and the anatomical structure was considered. Then a subsequent boost of 15–20 Gy was given to GTV plus a 0.5–1 cm margin. The dose of the boost is based on each physician's determination. All patients were followed up brain images and clinical visits regularly after therapy. This study was approved by our institutional review board and ethics committee.

Statistical analysis

OS and progression-free survival (PFS) were analyzed in both groups. OS censored patients at death or the date of last follow-up, and PFS documented locoregional recurrence or death from any cause. We applied Kaplan-Meier analyses for comparisons of survival and evaluated differences between the survival curves by the log-rank test. Differences in continuous variables were evaluated by the student *t* test or Wilcoxon rank sum test, and those in categorical variables were evaluated by the Fisher's exact test. To identify independent factors of survival, we used multivariate Cox proportional hazards models that included factors with p values smaller than 0.10 in the univariate analyses. Hazard ratios (HR) and 95% confidence intervals (CI) were listed. A two-tailed p value <0.05 was considered statistically significant. All analyses were conducted by SPSS package.

Results

From 2005 through 2016, 142 GBM newly diagnosed patients were identified in our database of cancer registry, and 84 of them fit in inclusion criteria. Of the 58 ineligible GBM patients, 52 did not undergo radiotherapy, 4 received palliative brain irradiation (< 50 Gy), and 2 were younger than 20 years old. The patient's age ranged from 21 to 84 years old (median, 61 years) at diagnosis (Table 1). Of 84 patients, 52 (62%) were ~~man~~ male, 27 (32%) were diagnosed before 2010. Most (63, 75%) of the patients underwent subtotal or GTR. and 82% received chemotherapy. Among the 69 (82%) patients who received chemotherapy, 46 (67% of the total) were prescribed a TMZ-based regimen. The radiation dose ranged from 50.0 Gy to 66.6 Gy with a median of 60.0 Gy. While, 15 (18%) patients received dose escalation boost >60.0 Gy with a median of 66 Gy (range 61.2 - 66.6 Gy), only four patients did not reach the full 66-Gy boost dose, but, no explicit medical record could be obtained. The irradiation volume ranged from 56 to 817 mL (median 247 mL). The median OS of all enrolled patients was 14.0 months, while the median PFS was only 9.0 months.

Table 1 Patient characteristics of glioblastoma

Characteristics	Patients (N =84) (n, %)
Age (years) (median, range)	61 (2–84)
Overall survival (month) (median, range)	14.0 (2.5–109.8)
Progression-free survival (month) (median, range)	9.0 (1.4–61.4)
Sex	
Male	52 (62)
Female	32 (38)
Operation[a]	
Yes	63 (75)
Biopsy	21 (25)
Radiation dose (Gy) (mean, range)	60.0 (50.0–66.6)
> 60	15 (18)
≤ 60	69 (82)

Radiation volume (mL) (median, range)[b]	247 (56–817)
Chemotherapy	
Yes	69 (82)
No	15 (18)
Temozolomide-based regimen	
Yes	46 (67)
Non-temozolomide regimen	23 (33)
Year of diagnosis	
2005—2010	27 (32)
2010–2016	57 (68)

[a]Subtotal or gross-total resection, other than biopsy only.
[b]27 patients did not have detailed radiation volume.

The 15 patients with dose-escalation (> 60 Gy) (escalated group) were younger than the 69 patients received the standard 60-Gy dose (reference group) (median, 52 *vs.* 62 years old, p =0.05), but the differences in age, sex, surgery, chemotherapy, and radiation volume did not reach statistical significance (Table 2). Following age stratification at 70 years, we found patients older than 70 years seemed to be more likely to receive the standard dose, but the difference did not reach statistical significance (26% *vs.* 7%, p =0.17). Following stratification of the year of diagnosis at 2010, we found a higher percentage of patients were assigned to receive the escalated dose before 2010 (73% vs. 27%, p <0.01).

Table 2 Comparison of glioblastoma patients treated with dose escalation (> 60 Gy) and standard dose (≤ 60 Gy)

Characteristics	Dose escalation (N, %)	Standard (N, %)	p value[a]
Patients number	15	69	
Overall survival (month) (median, range)	33.8 (6.2–109.8)	12.5 (2.5–77.2)	< 0.001
Progression-free survival (month) (median, range)	15.4 (13.1– – 61.4)	7.9 (1.4–55.0)	0.01
Age (years) (median, range)	52 (29–77)	62 (21–84)	0.05
Age group (year)			0.17
> 70	1 (7)	18 (26)	
≤ 70	14 (93)	51 (74)	
Sex			0.24
Male	7 (47)	45 (65)	
Female	8 (53)	24 (35)	

Surgery[b]			0.51
Yes	10 (67)	53 (77)	
biopsy	5 (33)	16 (23)	
Chemotherapy			0.73
Yes	12 (80)	57 (83)	
No	3 (20)	12 (17)	
Temozolomide-based regimen[c]			0.20
Yes	5 (56)	41 (69)	
Non-temozolomide regimen	4 (44)	13 (31)	
Year of diagnosis			<0.01
2005-2010	11 (73)	16 (23)	
2010-2016	4 (27)	53 (78)	
Radiation volume (mL) (median, range)[d]	247 (94–817)	249 (56–731)	0.66

[a]*p* value for student t test, Wilcoxon rank sum or Fisher's exact test.
[b]Subtotal or gross-total resection, other than biopsy only.
[c]6 patients did not have detailed records of chemotherapy regimen.
[d]27 patients did not have detailed records of radiation volume.

The escalated group survived a better PFS than the reference group, with a median of 15.4 (13.1 – 61.4) *vs.* 7.9 months (1.4 - 55), respectively (p =0.01 for log-rank test) (Fig 1). Besides, the escalated group survived longer, with a median OS of 33.8 months (range 6.2–109.8), in contrast to the reference group with 12.5 months (range 2.5–77.2) (p <0.01 for log-rank test) (Fig 2).

Fig 1. Kaplan-Meier estimates of progression-free survival comparing glioblastoma patients receiving the standard dose (≤60 Gy) and those receiving dose escalation (> 60 Gy)

Fig 2. Kaplan-Meier estimates of overall survival comparing glioblastoma patients receiving a standard dose (≤60 Gy) and those receiving dose escalation (> 60 Gy)

A univariate analysis to identify the factors affecting PFS found significant association with the escalated dose (hazard ratio [HR] =0.45, 95% confidence interval [CI]: 0.20–1.00, p =0.05), whereas age >70 years stratification (HR =1.64, p =0.07) seemed to have the trend to affect PFS. After a multivariate analysis, only the escalated

dose was identified as a significantly predictor for good prognosis (HR =0.43, p =0.03) (Table 3).

Table 3 Univariate and multivariate Cox regression analyses of progression-free survival for glioblastomas

Variable	Univariate HR (95% CI)	p-value	Multivariate[a] HR (95% CI)	p-value
Age group				
≤ 70	(reference)			
> 70	1.64 (0.95–2.84)	0.07	1.39 (0.79–2.43)	0.25
Sex				
female	(reference)		-	-
Male	1.12 (0.70–1.82)	0.63	-	-
Radiation dose				
≤ 60 Gy	(reference)			
> 60 Gy	0.45 (0.20–1.00)	0.05	0.43 (0.20–0.91)	0.03
Irradiation volume[b]				
< 250ml	(reference)			
≥ 250ml	0.62 (0.35–1.09)	0.11		
Surgery[c]				
No	(reference)		-	-
Biopsy	0.85 (0.51–1.44)	0.55	-	-
Chemotherapy				
No	(reference)		-	-
Yes	1.13 (0.59–2.16)	0.71		
Year of diagnosis				
2005-2010	(reference)			
2010-2016	1.37(1.12–3.32)	0.20		

HR, hazard ratio; CI, confidence interval.
[a]p value <0.10 were included in multivariate analysis.
[b]27 patients did not have detailed radiation volume.
[c]Subtotal or gross-total resection, other than biopsy only.

A univariate analysis showed only two factors affected OS. The escalated group had a better survival (HR =0.34, p <0.01), and those diagnosed after 2010 experienced a shorter survival (HR =2.01, p <0.01). The other variables did not affect OS, including age stratification, gender, surgery, prescribed chemotherapy or not, and radiation volume. Further, two factors (diagnosis year and radiation dose) were put into analysis of the multivariate Cox proportional hazard model. Only the escalated dose was identified as a predictor for better prognosis (HR =0.40, p < 0.01) (Table 4).

Table 4 Univariate and multivariate Cox regression analyses of overall survival for glioblastomas

	Univariate		Multivariate[a]	
Variables	HR (95% CI)	*p*-value	HR (95% CI)	*p*-value
Age group				
≤ 70	(reference)			
>70	1.29 (0.76–2.20)	0.35		
Sex				
female	(reference)		-	-
Male	1.18 (0.74–1.86)	0.49	-	-
Radiation dose				
≤ 60 Gy	(reference)			
> 60 Gy	0.34 (0.18–0.64)	< 0.001	0.40 (0.21–0.78)	0.007
Radiation volume[b]				
< 250 mL	(reference)			
≥ 250 mL	0.75 (0.42–1.31)	0.30		
Surgery[#]				
Biopsy	(reference)		-	-
Yes	0.93 (0.55–1.58)	0.78	-	-
Chemotherapy				
No	(reference)		-	-
Yes	0.77 (0.43–1.38)	0.37		

Year of diagnosis				
2005-2010	(reference)			
2010-2016	2.01 (1.12–3.32)	0.006	1.52 (0.9 – 2.57)	0.12

HR, hazard ratio; CI, confidence interval.
[a] p value < 0.10 were included in multivariate analysis .
[b] 27 patients did not have detailed radiation volume.
[c] Subtotal or gross-total resection, other than biopsy only.

Discussion

GBM is the deadliest primary malignant brain tumor, typically presenting as a single brain tumor and tending to recur locally just adjacent to the primary tumor site. Despite improvements in imaging, surgery, radiotherapy, and chemotherapy, the majority of GBM patients continue to recur at the site of the original tumor site. According to previous studies on the pattern of failure for GBM patients, more than 80% of tumor recurred within 2 cm of the pre-surgical tumor margin. Local tumor progression or relapse remains within or adjacent to the original brain lesion in approximately 90% of GBM patients [3-5]. In regard to this situation, local control is most paramount to improve the GBM outcome. There are many clinical studies on the extent of surgery and escalated radiation dose to prevent local recurrence. However, clinical circumstances are often associated with the difficulties in achieving a GTR, such as old age, comorbidities, performance status, large tumor size, and multiple lesions [4, 6, 13-16].

Pooled analysis of six randomized trials of radiotherapy versus no radiotherapy following surgery reported significant survival benefits in the cohort of radiotherapy [17, 18]. However, the survival advantage following standard 60-Gy radiation was poor, and OS remained dismal without long-term survivors. Therefore, prescribing a higher radiation dose for GBMs has been attempted in many clinical studies, including altered

fractionation, stereotactic radiosurgery, and brachytherapy to deliver a higher dose to the PTV region in the hope of improving local tumor control. A clear dose-response relationship has not been identified for GBMs. However, our report did support the survival benefit of GBMs using the IMRT technique to deliver a moderately escalated dose (> 60-Gy) with conventional 1.8–2 Gy daily fractionation in contrast to standard 60-Gy regimen only. Our report is well in line with a dose escalation study of Zschaeck et al [11]. The study reported 133 patients received standard 60-Gy radiotherapy, while 23 received moderate dose escalation (66-Gy) and found a higher dose lead to improve intracranial recurrence, which translated to better OS than the 60-Gy group. Zhong et al. also reported the improved PFS and OS following a moderately integrated radiation boost scheme (64 Gy over 27 fractions to GTV) in GBM patients [12]. Graf et al. also reported the primary tumor control rate was correlated with the irradiation dose. The median survival time was 3.0 months for a radiation dose of 55 Gy or less, 8.6 months for doses between 56 and 65 Gy, and 9.6 months for a dose range between 66 and 75 Gy (p< 0.01) [19]. Another two studies also showed a survival benefit compared to historical data in patients that received a higher radiation dose [20, 21]. Our data suggest an association of moderate radiation dose escalation with improved survival in our GBM patients.

On the contrary, the RTOG 98-03 study allocated GBM patients to accept four escalated radiation 66, 72, 78, and 84-Gy dose groups. No survival benefit was found among groups with dose escalation. However, the results only concluded the feasibility of delivering higher than standard 60-Gy with an acceptable toxicity of late brain necrosis [9]. Nakagawa et al. reported a trial of dose escalation up to 90 Gy, and found those receiving such a high dose had a lower rate of local recurrence, whereas the OS was similar to the conventional dose group. Though this study concluded the benefit of

a higher dose is not quite clear [10]. Some literature also reported that an escalated dose beyond 60 Gy for GBM did not improve OS [5, 9, 10, 22]. To conclude, the National Comprehensive Cancer Network still considered 60-Gy radiotherapy as the standard of treatment. The debate of the pros and cons of an escalated radiation dose in the GBM have continued. So, we conducted a literature review to identify the relationship between escalation and outcome. Databases of Medline and PubMed were searched. Clinical cases series and Radiation Therapy and Oncology Group reports are listed in Table 5.

Table 5 Literature review on the escalated radiation dose trial of glioblastoma

Study [reference]	Escalated radiation dose trial	N	Median survival (months)	Concurrent chemotherapy	Survival benefit
Nakagawa *et al.* (1998)[10]	Low dose (60–-80Gy)	21	17	Nimustine, vincristine	No
	High dose (90 Gy)	17			
RTOG 98-03(2002) [9]	PTV< 75 cm3 46 Gy in 23 fractions + 20 -38 Gy (3DCRT)	94	11.6 (66Gy)<br>11.8 (72Gy)<br>11.8 (78Gy)<br>19.3 (84Gy)	BCNU	No
	PTV≥ 75 cm3 46 Gy in 23 fractions + 20 -38 Gy (3DCRT)	109	8.2 (66Gy)<br>6.5 (72Gy)<br>6.9 (78Gy)<br>6.0 (84Gy		
Chan *et al.*(2002) [5]	60 Gy + 10 Gy (3DCRT)	20	13.9	No	No
	60 Gy + 20 Gy	55	12.9		
	60 Gy + 30 Gy	34	11.7		
RTOG 93-05(2004)[21]	60 Gy in 30 fractions	97	13.6	BCNU	No
	60 Gy + 15–24 Gy (stereostatic radiosurgery)	89	13.5		

Graf *et al.* (2005) [18]	56-65Gy	8.6	8.6	No	Yes
	66-75Gy	9.6	9.6		
Tsien *et al.* (2012)[20]	66—81Gy	38	20.1	TMZ	Yes
Zschaeck *et al.* (2018)[11]	60Gy	133	15.3	TMZ	Yes
	66Gy	23	18.8		
Kim *et al.* (2019) [19]	66-81Gy	82	18.7	TMZ	Yes
Zhong et al. (2019) [12]	64 Gy in 27 fractions (SIB)	80	21	TMZ	Yes
Current study	60 Gy in 30-33 fractions	69	12.5	TMZ	Yes
	>60 Gy (conventional boost)	15	33.8		

RTOG, radiation therapy and oncology group; SIB, simultaneous integrated boost; 3D CRT, 3D conformal radiotherapy; TMZ: temozolomide.

It is well-known GTR is a crucial management in GBM. However, GBM is a diffusely infiltrative brain tumor, which tumor cells preferably extend beyond the maximum resection. Therefore, GTR only could be regarded as a debulking operation, and, in the most clinical scenarios, extensive radical surgery is not feasible. Kreth et al. reported only about 50% of patients could receive GTR and have better survival [14]. Awad et al. found OS significantly improved with the extent of resection in a univariate analysis, averaging 22, 19, and 13 months for >90, 80–90, and 70–80% tumor resection, respectively. But after a multivariate analysis, the extent of resection no longer correlated with OS [22]. Our study only differentiated between tumor resection and biopsy-only from hospital database analysis, and found no significant association between resection and survival benefit. In the clinical setting, radical surgery should be weighted between the clinical recovery and risk of neurologic deficit. Although we failed to demonstrate the benefits of the extent of resection in our cohort, undoubtedly, we believe the extent of resection is imperative in GBM. Nonetheless, our analysis did not identify survival benefit in GBMs with subtotal or gross-total resection.

We analyzed several prognostic variables including age, gender, radiation volume, and chemotherapy. The date of diagnosis before 2010 was a significant factor for worse OS in a univariate analysis. However, after adjusting the radiation dose, it was no longer to impact OS in the multivariate analysis. The number of GBM patients assigned to accept dose escalation with conventional boost had decreased after date of diagnosis after 2010 in our hospital. The reason opted to standard 60-Gy either dose intensification may come with a cost, or dose intensification has involved to various techniques of hypofractionated and simultaneous integrated boost dose, and/or stereotactic radiosurgery boost [23, 24].

The age of escalated dose was younger than standard group in the study. Younger

age is significantly associated with good survival in GBM patients in the literature [24, 25]. So a relevant chance of hidden bias of age factor should be cautious in our study. Evidence-based clinical practice guidelines recommend patients older than 70 years old consider hypofractionated radiotherapy [26]. So we categorized the cutoff age to 70 years, and found the age > 70 stratification had a trend to impact PFS in an univariate analysis, and, however, after adjusting the radiation dose, it was no longer to impact PFS in multivariate analysis. Roa et al. reported on GBM patients aged 60 years or older being randomly assigned after surgery to receive either standard radiation (60 Gy in 30 fractions over 6 weeks) or a shorter course of radiation (40 Gy in 15 fractions over 3 weeks). There was no difference in survival between patients receiving standard and short-course radiation [26].

Currently, GBM patients receive adjuvant TMZ as the standard chemotherapy regimen. Nonetheless, only 46% of our patients received TMZ regimen, and chemotherapy dose and regimen did not conform in our study. The reason was due to TMZ not being fully reimbursed by the National Health Insurance in Taiwan in the early years of 2000s. Therefore, some patients were treated with various chemotherapy regimens, like BCNU and cisplatin. Non-uniform chemotherapy might mask the therapeutic significance in the study.

The 2016 WHO classification of central nervous tumors introduced molecular parameters in addition to histology to define GBM tumor entities [27]. However, we enrolled GBM patients between 2005 and 2016, and the marker of IDH mutation and MGMT were not mandatory for routine pathologic reports. We are therefore unable to analyze the prognostic significance among these molecular markers in our cohort.

Conclusions

Although the current study was limited by its retrospective design, the escalated dose (> 60-Gy) remained survival benefit after adjusting for other prognostic variables. However, there are major shortcomings of our study, e.g. non-uniform chemotherapeutic treatment and younger patients in the high dose group. These and the general restrictions of a retrospective analysis limit implications of this analysis. Moreover, the molecular characteristics, such as MGMT or IDH mutation status, were not routinely assessed in the cohort. A prospective randomized trial is needed to confirm results of this study.

Acknowledgements

The authors thank the Department of Cancer Registry of Chi Mei Medical Center, Tainan, Taiwan and Chi-Mei Hospital, Liouying, Tainan, Taiwan for their assistance to this study.
