# Supplementary material for: Survival of glioblastoma treated with a moderately escalated radiation dose—Results of a retrospective analysis
Source: PLoS One. 2020 May 15;15(5):e0233188. doi: 10.1371/journal.pone.0233188 (PMC7228055; doi:10.1371/journal.pone.0233188)
Supplement: Attachment — Submitted filename: Response_to_reviewers_PONE_D-20-02836.docx [file pone.0233188.s004.docx]

**Response to Reviewers’ Comments**

First, we feel very happy to receive substantive and excellent comments from reviewers. We respond the comments line by line accordingly. The revised manuscript was marked in red in corrected words, paragraphs, and sections.

**Comments from Reviewer 1：**

1. While the authors acknowledge the lack of molecular characterization, the absence of capturing/adjusting for IDH/MGMT severely limits interpretation of clinical outcomes in a small cohort. If at all possible I would encourage the authors to adjust for either of the above even if many values are missing.

***Response:***

The 2016 WHO classification of central nervous tumors has introduced molecular parameters in addition to histology to define GBM tumor entities. However, we enrolled GBM patients between 2005 and 2016, and so the markers of IDH mutation and MGMT were not mandatory in routine pathologic reports. Actually, in our cohort, we could not find any one who had been checked for the status of MGMT. In response to the Reviewer’s comment, we changed the last sentence of Discussion to “Because of the lack of data, we were unable to evaluate the prognostic significance of these molecular markers in our study.” in the revised manuscript. We have also added this as a limitation to Discussion and Conclusion in the revised manuscript.

 2. I would encourage the authors to consider using statistical modeling such as penalized Cox models with a LASSO selector given the small cohort and relatively large number of factors of interest. More robust methodology would further limit the potential for multiple comparison error.

***Response:***

According to the Reviewer’s suggestion, we performed the analysis and added the results of LASSO approach in Table 3, Table 4, and Supplement Table 1. Following detailed statistical analysis, a moderate radiation dose escalation (> 60 Gy) was still identified as an independent factor affecting OS in GBM patients. In addition, we have added related description to the Abstract and main text in the revised manuscript.

3. Rather than adjusting for chemotherapy/non-chemotherapy, I would encourage the authors to adjust for temozolomide/non-tmz chemo/none given the substantial improvement with TMZ in MGMT methylated patients (perhaps to some extent in unmethylated patients as well given the potential for protein expression despite methylation status).

***Response:***

Among the 69 (82%) patients who received chemotherapy, only 46 (67% of the total) were prescribed with a TMZ-based regimen. In response to the Reviewer’s comments, we have tried to make comparison among three groups, namely temozolomide chemotherapy, non-TMZ chemotherapy, and no chemotherapy, but found no significant differences regarding overall survival (OS). We have added the result in Table 4.The results are shown in following table:

|  | Univariable (OS) |  |
| --- | --- | --- |
| Variable | HR (95% CI) | *p*-value |
| No chemotherapy | (reference) | 0.211 |
| TMZ-based regimen | 0.56(0.30-1.08) |  |
| Non-TMZ Chemotherapy | 0.54(0.18-1.60) |  |

**4.** Would recommend the authors mention NRG-BN-001 which is investigating dose-escalated RT for patients with GBM in the era of TMZ.

***Response:***

According to the Reviewer’s suggestion, we have added related information to Discussion in the revised manuscript.

**5.** It is a little concerning to me outcomes were worse for patients diagnosed after 2010. Given multiple large database analyses have associated improved OS in more modern cohorts (PMID 27214765, 28452053) this finding to me highlights the potential presence of an unadjusted maldistribution of molecular mutations (IDH/MGMT) which may be modulating PFS/OS.

***Response:***

We can understand the Reviewer’s concern that the survival became worse in patients who received treatment in recent years. Although we have no data to address the possible effects of IDH/MGMT, we believe it was quite likely attributable to dose escalation. Most of the patients who received the escalated dose were diagnosed before 2010, while most of those who received the standard dose were diagnosis after 2010. We have added related description to Discussion in the revised manuscript.

**Comment from Reviewer 2：**

This retrospective study evaluated the effect of radiation dose on survival outcomes for patients with glioblastoma and identified dose escalation as being associated with improved outcomes. The authors' study adds to the literature regarding dose escalation. Since GBM frequently recurs locally, it is important to identify factors that correlate with local recurrence. Although the dose escalation group only included 15 patients, it is interesting that these patients did experience improved OS. Only one patient in this group was over the age of 70, while there were 18 patients over age 70 in the standard dose arm. Although age was found to not be associated with outcomes on multivariate analysis, it is probable that this would be significant with a larger sample size since age is so critical to GBM outcomes. One recommendation for the authors is to more clearly separate which patients underwent GTR or STR, since completeness of resection is also associated with improved outcomes in this patient population. Additionally, I recommend more clearly highlighting that there was no difference in treatment volume between the two arms. Overall, this is an interesting study, and although retrospective in nature with all the requisite caveats and without molecular data, it adds an important data point to GBM literature with respect to radiation dose.

***Response:***

We thank the Reviewer for the encouraging comment. While we agree that the completeness of resection is also associated with improved outcomes, because of the small case number, we would not have the statistical power to adjust for more variables. We added “the completeness of resection is also associated with improved outcomes, because of the small case number, we could not evaluate or adjust its effects in our study” as a limitation to both Discussion. As suggested by the Reviewer, we also added “but their treatment volumes were similar to the others” to the Abstract and “with similar treatment volumes” to the Discussion to highlight that there was no difference in treatment volume between the two arms.

**Comment from Reviewer 3：**

Overall the manuscript is logical and states its purpose. The subject is not original, but the data and the patients cohorts reported in the study are unique, thereby meeting one of the criteria for publishing in PLOS One. It would be good if there were more patients, and I would encourage the authors to see if they can combine patient cohorts with other centers, recognizing that this may not ultimately be possible. There are, however, many grammatical and work usage errors in the manuscript, and at a minimum these need to be corrected. I am scanning the manuscript with my copyedits in blue.

***Response:***

We agree with the Reviewer’s suggestion that a multicenter study is a good idea to expand the database, but it is not achievable in a short period time. We believe the publication of this manuscript will provide a more solid ground to call for such a study. We really appreciate the editing of the manuscript and have corrected those and other errors in the manuscript.
